# Chondrogenic differentiation of Wharton’s Jelly mesenchymal stem cells on silk spidroin-fibroin mix scaffold supplemented with L-ascorbic acid and platelet rich plasma

**DOI:** 10.1038/s41598-020-76466-8

**Published:** 2020-11-10

**Authors:** Anggraini Barlian, Hermawan Judawisastra, Ahmad Ridwan, Antonia Ratih Wahyuni, Meidiana Ebtayani Lingga

**Affiliations:** 1 School of Life Science and Technology, Bandung Institute of Technology, Bandung, West Java 40132 Indonesia; 2 Research Center for Nanosciences and Nanotechnology, Bandung Institute of Technology, Bandung, West Java 40132 Indonesia; 3 Faculty of Mechanical and Aerospace Engineering, Bandung Institute of Technology, Bandung, West Java 40132 Indonesia

**Keywords:** Biotechnology, Cell biology, Stem cells, Materials science

## Abstract

In this research, hWJ-MSCs were grown on silk scaffolds and induced towards chondrogenesis by supplementation with L-ascorbic acid (LAA) or platelet rich plasma (PRP). Silk scaffolds were fabricated with salt leaching method by mixing silk fibroin (SF) with silk spidroin (SS). The silk fibroin was obtained from *Bombyx mori* cocoon that had been degummed, and the silk spidroin was obtained from wild-type spider *Argiope appensa*. The effect of scaffold composition and inducer on cell proliferation was observed through MTT assay. The most optimal treatment then continued to be used to induce hWJ-MSC towards chondrogenic differentiation for 7 and 21 days. Scaffolds characterization showed that the scaffolds produced had 3D structure with interconnected pores, and all were biocompatible with hWJ-MSCs. Scaffold with the addition of 10% SS + 90% SF showed higher compressive strength and better pore interconnectivity in comparison to 100% silk fibroin scaffold. After 48 h, cells seeded on scaffold with spidroin and fibroin mix had flattened morphology in comparison to silk fibroin scaffold which appeared to be more rounded on the scaffold surface. Scaffold with 10% (w/w) of silk spidroin (SS) + 90% (w/w) of silk fibroin (SF) was the most optimal composition for cell proliferation. Immunocytochemistry of integrin β1 and RGD sequence, showed that scaffold with SS 10% provide better cell attachment with the presence of RGD sequence from the spidroin silk which could explain the higher cell proliferation than SF100% scaffold. Based on Alcian Blue staining and Collagen Type II immunocytochemistry (ICC), cells grown on 10% SS + 90% SF scaffold with 10% PRP supplementation were the most optimal to support chondrogenesis of hWJ-MSCs. These results showed that the addition of spidroin silk from *A. appensa*. had impact on scaffold compressive strength and chondrogenic differentiation of hWJ-MSC and had the potential for further development of bio-based material scaffold in cartilage tissue engineering.

## Introduction

Articular cartilage is tissue that lacks vascular, nervous, and lymphatic systems, with chondrocytes located sparsely between the matrix. These properties limit tissue regeneration once damage or injury occurs. Therefore, the tissue does not have the capacity to be fully repaired^1,2^. Tissue engineering is currently studied as a new alternative for articular cartilage damage. It has three important, mutually interacting factors that regenerate or repair the damaged tissue: cells, biomaterial/scaffold, and bioactive factors^3,4^. Mesenchymal stem cells (MSC) are currently being studied as a new therapy to repair damaged articular cartilage. MSC can be isolated from bone marrow, adipose tissue, or Wharton’s Jelly (WJ) tissue from the umbilical cord^5^. The process for isolation of cells from the umbilical cord does not cause problems in terms of ethical consideration^6^. MSCs derived from hWJ have wider plasticity, and a higher proliferation rate compared to other MSCs cell sources^7^. hWJ-MSCs are widely used in the development of tissue engineering to differentiate into chondrocyte, and are able to increase the production of hyaluronic acid and glycosaminoglycans as well as collagen type II, the specific component of articular cartilage ECM^8^. Platelet rich plasma (PRP) and ascorbic-2-phosphate acid (LAA) are bioactive factors that control MSC towards chondrogenic differentiation. PRP is an autologous derivative of blood tissue that mainly contains important growth factors, such as transforming growth factor, platelet-derived growth factor, and epidermal growth factor, that are important for cell proliferation and chondrogenic differentiation^9,10^. LAA is able to increase the proliferation rate of cells derived from mesenchymal and to support differentiation. LAA is also an important cofactor for the key enzyme in collagen biosynthesis, the latter is part of the extracellular matrix^11^. Scaffold in tissue engineering is important for providing structure and substrate for cells to proliferate and undergo differentiation^12^. Methods for producing porous 3D scaffolds can be achieved through salt leaching, 3D-printing, or emulsion freeze drying. Producing scaffolds with pores of a certain size can be achieved by the salt leaching method^13^. Currently, silk has been studied in tissue engineering for its biocompatibility and biodegradability^14^. Silk fibroin (SF) produced by *Bombyx mori* has been extensively studied as biomaterial for articular cartilage and bone tissue engineering since it has high mechanical strength and low immunogenicity^15^. Previous research has shown that silk fibroin scaffold was able to support chondrogenic differentiation of adipose derived stem cells (hADSC)^16^. However, cells are known to attach weakly to fibroin scaffold due to the lack of specific amino acid sequence recognized by cells^17,18^. Silk spidroin (SS) produced by spider *A. appensa*. has never been studied before. Silk spidroin produced by spiders is well known for its mechanical strength and non-toxicity^19^. In this research, silk fibroin was mixed with silk spidroin to produce 3D porous scaffold that could improve hWJ-MSCs attachment, proliferation, and chondrogenic differentiation. Chondrogenic differentiation of cells on the silk scaffolds were induced by the addition of PRP or LAA in the medium.

## Results and Discussion

### FTIR spectroscopy of the silk fibroin-spidroin fiber and scaffold

**Figure 1:**
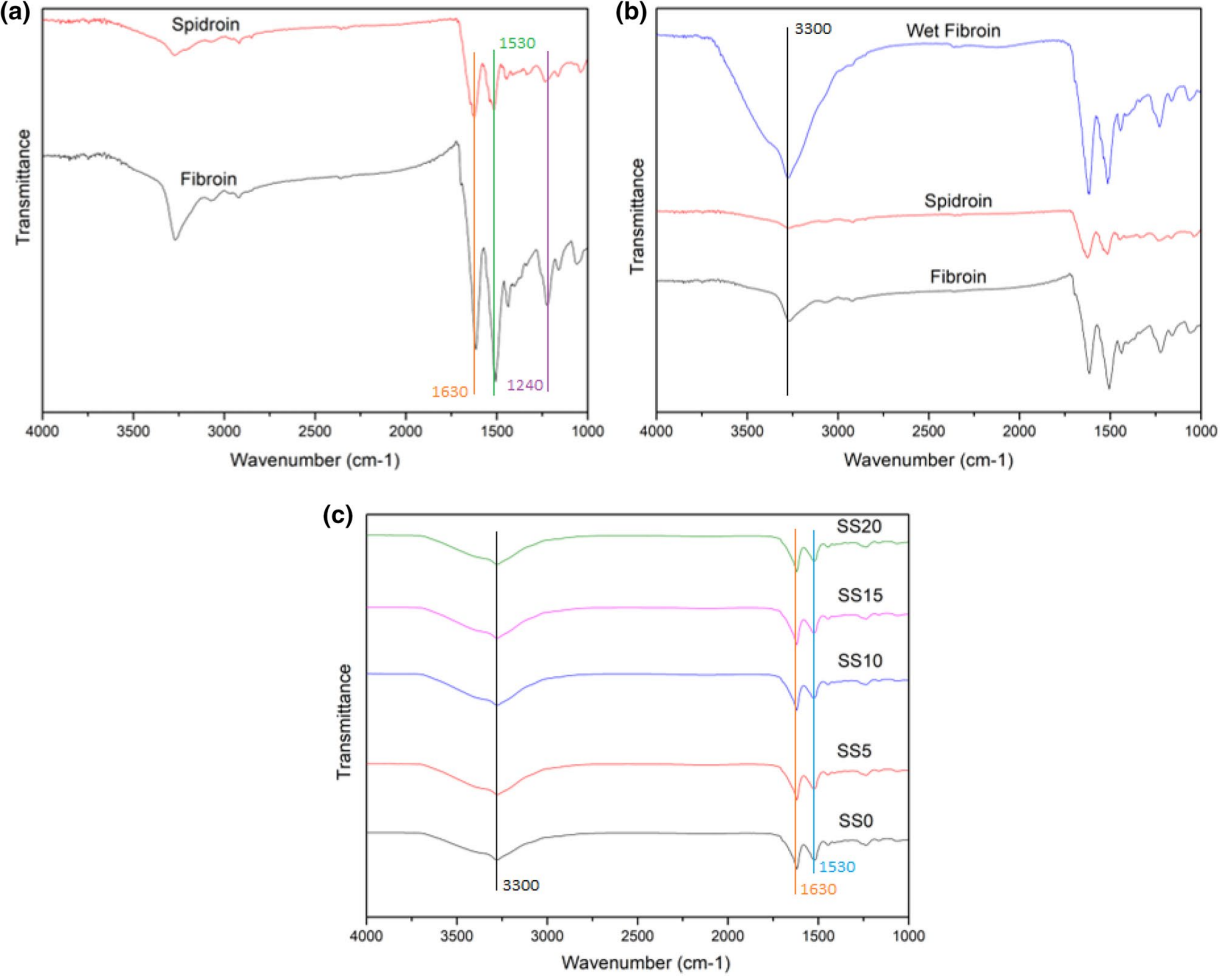
FTIR analysis of (**a**) silk spidroin and fibroin fiber, (**b**) wet silk fibroin in comparison to dry silk fibroin and spidroin, and (**c**) various composition of silk fibroin and spidroin-mix scaffolds. SS0 = SF100%, SS5 = SF95% + SS5%, SS10 = SF90% + SS10%, SS15 = SF85% + SS15%, SS20 = SF80% + SS20%.

FTIR analysis was performed to the unprocessed silk spidroin (SS) and fibroin (SF) (Fig. 1a,b) and the processed scaffolds (Fig. 1c). Amide I can be observed on 1513 cm^−1^ wavelength, amide II on 1230 cm^−1^, and amide III on wavenumber around 1440 cm^−1^^20^. Silk fibroin is known to have specific wavenumber for β-sheet at 1630, 1530, and 1240 cm^−1^. Wavenumber at around 3300 cm^−1^ is specific for hydrogen bonds^21,22^. FTIR result of unprocessed silk fibroin and spidroin showed that unprocessed silk spidroin had slightly lower amide transmittance in comparison to silk fibroin. Wet unprocessed silk fibroin fiber was also compared to dry spidroin and fibroin, the result showed that water had effects on wavenumber at 3300 cm^−1^ which correspond to hydrogen bonds. On the processed scaffold, it can be observed that salt leaching process had slight effect on widening at wavelength around 3300 cm^−1^. Overall, the incorporation of silk spidroin into each scaffold composition had no effect on amide bonds and secondary structure of the protein.

### Scaffold contact angle and wettability measurement

**Figure 2:**
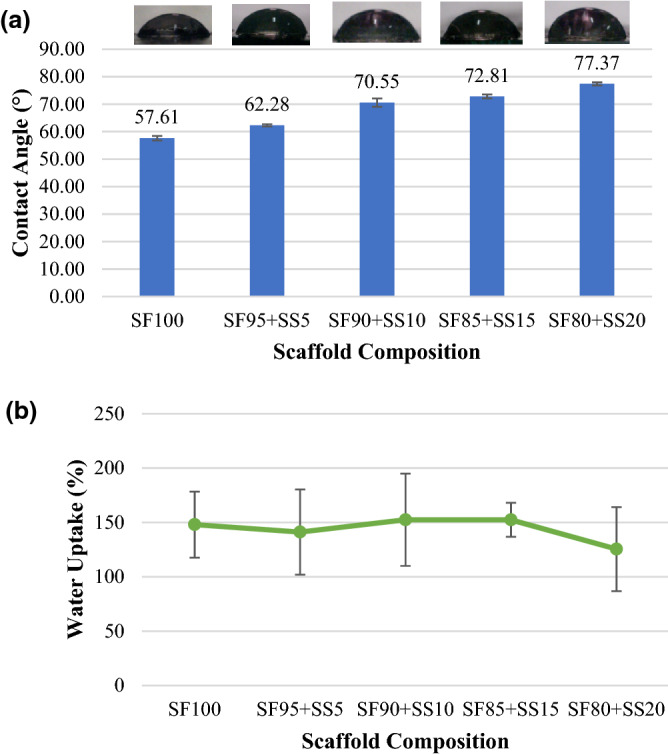
(**a**) Contact angle and (**b**) water uptake measurement of the scaffolds with various silk fibroin and spidroin compositions. SF = silk fibroin; SS = silk spidroin.

Contact angle and water uptake measurements were performed to assess the hydrophilicity of the scaffolds. In tissue engineering, hydrophilic biomaterial could facilitate better cell adhesion The ideal contact angle for a biomaterial to be hydrophobic would be under 90°^23^. Previous study on silk fibroin scaffold with direct dissolution technique showed that 100% silk fibroin scaffold (12% w/v) contact angle was 58°, which was around the result obtained in this study (Fig. 2a) at 57,61 ± 0,83°^24^. The mixing of silk spidroin into silk fibroin to form a scaffold had increased the contact angle. Biomaterial ability to absorb water or water uptake is also an important properties in tissue engineering to enable tissue regeneration and repairing^25^. The result from water uptake measurement (Fig. 2b) showed similar water uptake capacity between silk fibroin scaffold and scaffold with silk spidroin (125,433% to 141,177%), except for scaffold composition SF 80% + SS 20% (125,453%). Silk fibroin was known to have hydrophilic amino acids and carboxylic group, therefore it was able to improve hydrophilicity when combined with hydrophobic biomaterials such as polycaprolactone (PCL)^26^. Scaffold with the addition of 20% silk spidroin had lower hydrophilic properties and water uptake capacity, which could be influenced by the amino acid composition of silk spidroin and eventually tertiary structure of the protein.

### Scanning electron microscope (SEM) analysis of silk fibroin-spidroin mix scaffold

**Figure 3:**
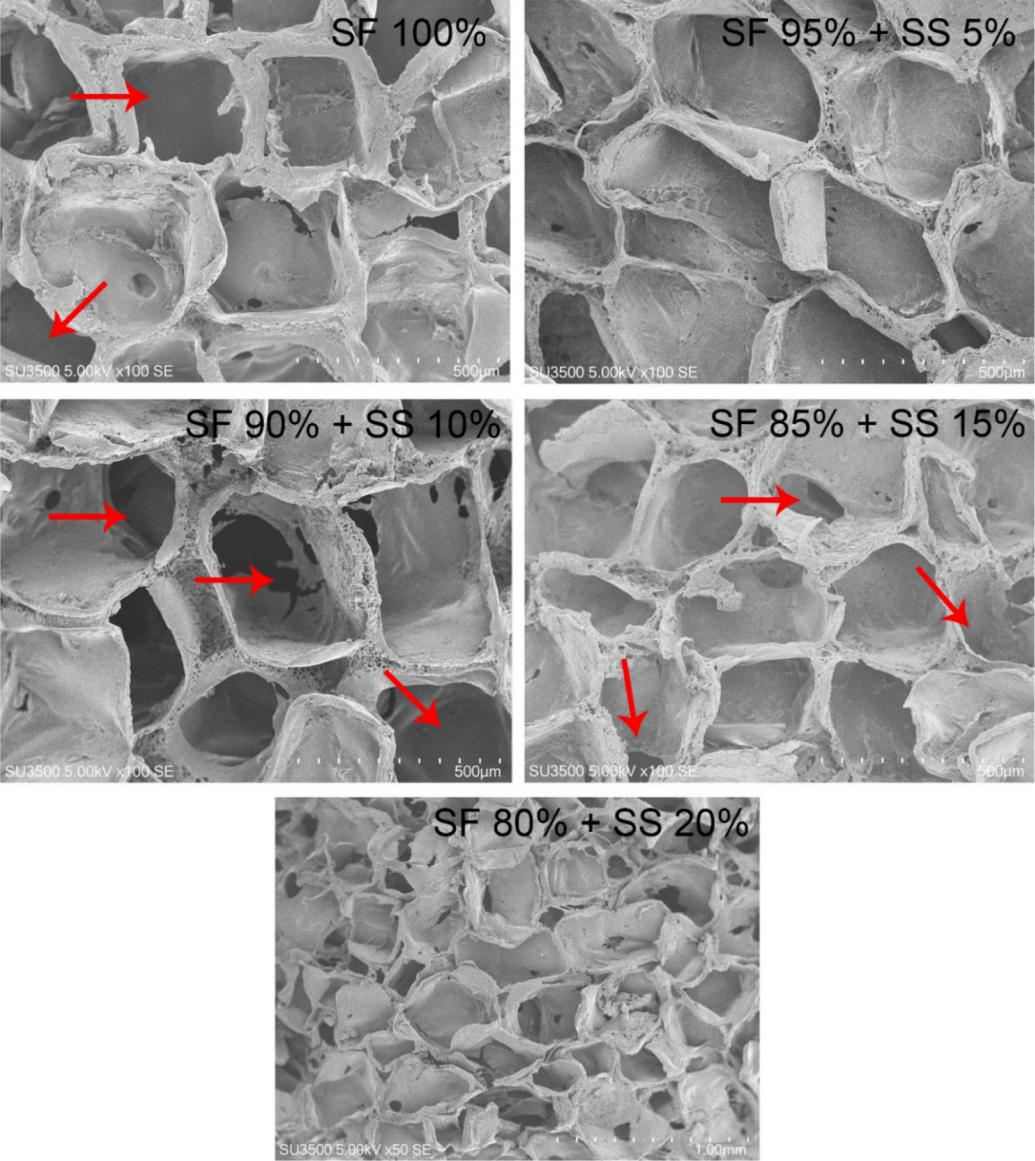
Morphology of pore interconnectivity formed on scaffold with the following composition: SF 100%, SF 95% + SS 5%, SF 90% + SS 10%, SF 85% + 15%, dan SF 80% + SS 20%. SF = silk fibroin, SS = silk spidroin. Red arrows show the formation of pore interconnectivity.

The pore size and interconnectivity formed on the silk scaffolds were observed with SEM (Fig. 3). Formation of pores was observed in all scaffold variations, but not in SF 80% + SS 20%; while the formation of interconnected pores could be observed in all scaffold variations, except for SF 95% + SS 5% scaffold. The average pore size of the scaffolds was measured using ImageJ software (Table 1). The overall pore size formed on scaffold was between the range of NaCl particles used (400–570 μm). Several studies have reported that scaffold with larger pore size; 370–400 μm^27^, 400–500 μm^16,28^ were able to support MSCs chondrogenesis better than smaller pore size, mainly due to higher permeability of gases and nutrition from culture medium^29^.

**Table 1:** Average pore size formed on silk scaffold produced using salt leaching method.

NaCl size (μm)	Scaffold composition			
	SF 100%	SF 95% + SS 10%	SF 90% + SS 10%	SF 85% + SS 15%
500 ± 21	462 ± 65	477 ± 53	456 ± 61	412 ± 79

*SF* silk fibroin; *SS* silk spidroin.

### Scaffold compressive strength

**Figure 4:**
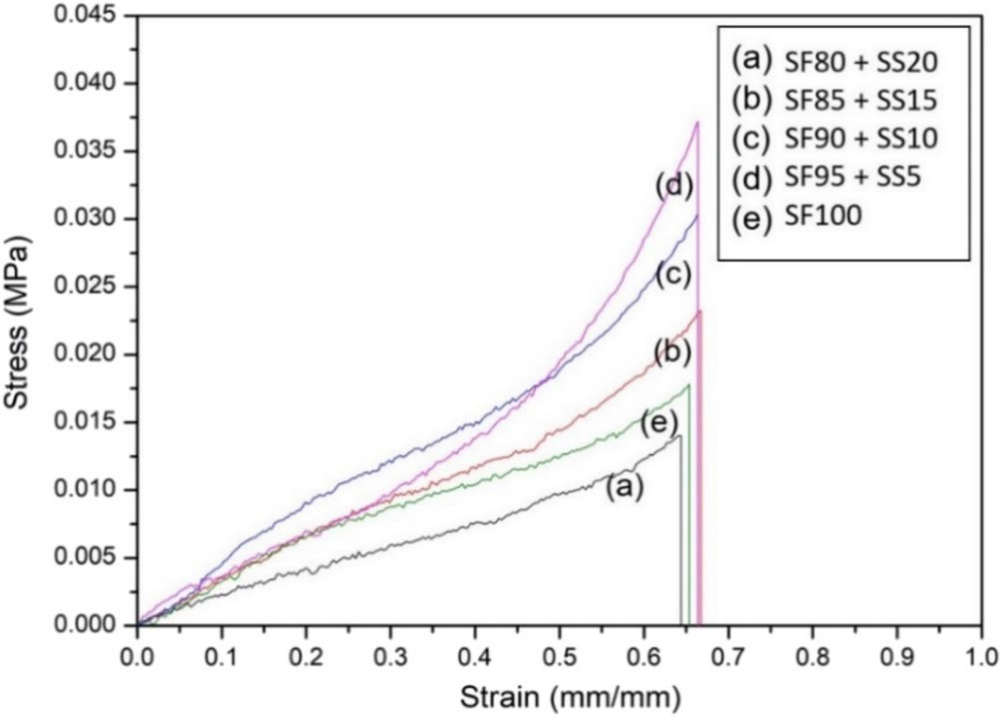
Stress–strain curve of several compositions of silk fibroin (SF) and spidroin (SS) mix scaffold in comparison to 100% silk fibroin scaffold. SF = silk fibroin; SS = silk spidroin.

Mechanical properties of the scaffolds with different silk fibroin and silk spidroin composition were observed by measuring compressive strength. In Fig. 4, scaffold with the addition of 5%SS and 10% SS had the highest compression strength, measuring at 0,0372 MPa and 0,0304 MPa respectively, while SF 100% and scaffold with 20% had the lowest mechanical strength. Based on these observations, the addition of silk spidroin into the scaffolds had effect in improving the compressive strength. However, the increase was dependent on silk spidroin concentration since scaffold with the highest silk spidroin concentration (SF80% + SS20%) had the lowest mechanical properties. Compressive strength of porous scaffolds is influenced by material composition, pore diameter, and porosity^30^. Incorporation of silk spidroin which is known for its mechanical strength were able to improve compressive strength of the scaffolds in this study^19^. Healthy cartilage is known to have compressive modulus between 0,24–0,85 MPa, and several engineered constructs are known to have modulus between 0.005–5.9 MPa^31^. Scaffold with 5% of SS were better in mechanical strength than scaffold with 10%SS, however for further study the SF90% + SS10% scaffold would be used as it had more ideal pore morphology with interconnectivity which would be ideal to support cell growth and differentiation.

### hWJ-MSC isolation and characterization

**Figure 5:**
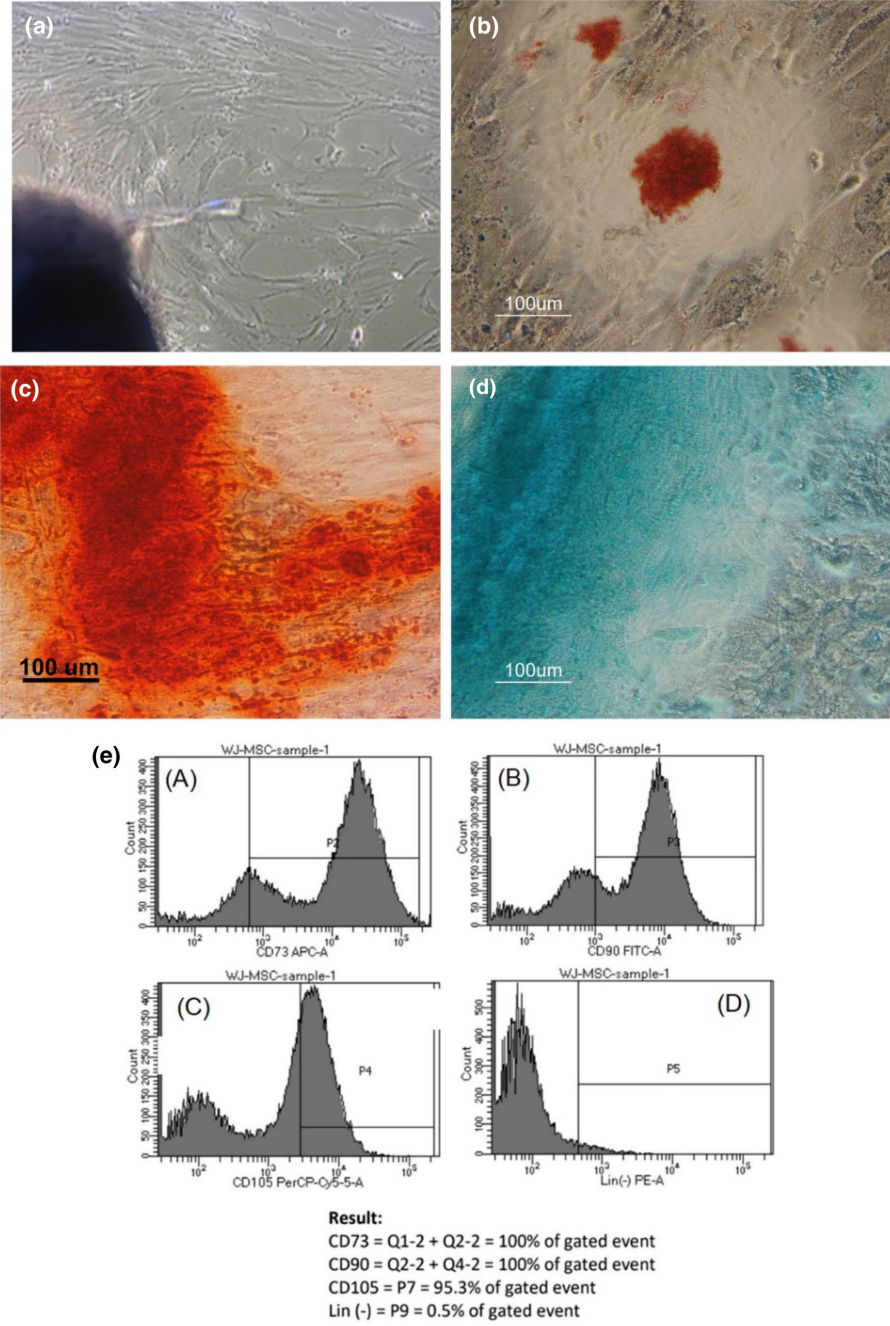
(**a**) Morphology of primary hWJ-MSCs migrated from WJ explant after 12 days of culture. Multipotency assay staining: (**b**) adipogenic differentiation stained with Oil Red O adipogenic differentiation; (**c**) Osteogenic differentiation stained with Alizarin Red; (**d**) Chondrogenic differentiation stained with Alcian Blue. (**e**) Analysis of positive MSC specific surface marker CD73 (100%), CD90 (100%), CD105 (95.3%), and Lin(-) negative marker (0.5%).

hWJ-MSCs were isolated from the WJ tissue of umbilical cords from cesarean sections using explant method^32^. The morphology of the primary hWJ-MSCs that had migrated from the WJ explants (Fig. 5a) were fibroblast-like and adherent to tissue culture flask surface under a standard culture condition. Multipotency of the hWJ-MSCs was confirmed by induction with adipogenic, chondrogenic, and osteogenic medium for 21 days. Lipid droplet formation was confirmed with Oil Red O staining (Fig. 5b), GAG with Alcian Blue staining (Fig. 5c), and calcium accumulation with Alizarin Red staining (Fig. 1d). Flow cytometry analysis (Fig. 5e) showed that the isolated hWJ-MSCs were positive for these MSC markers: CD90 (100%), CD73 (100%), and CD 105 (95.3%) at percentages higher than 95% and negative for CD34, CD45, CD11b, and CD19 (0,5%) at percentages lower than 2%. Based on these characterization results, the isolated hWJ-MSCs were in accordance with MSC characteristics as stated by ISCT (International Society for Cellular Therapy)^33,34^.

### Morphology of hWJ-MSC seeded on silk fibroin-spidroin mix scaffold

**Figure 6:**
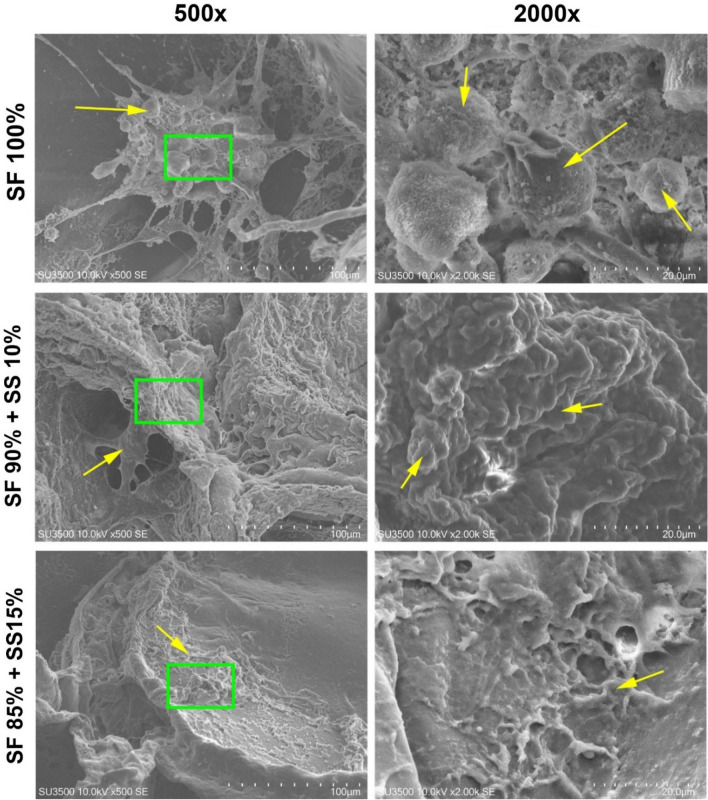
Morphology of hWJ-MSC grown on silk scaffold observed with scanning electron microscope (SEM) with 500 × dan 2000 × zoom. Green square shows the area zoomed at 2000x, and yellow arrow shows the cell morphology. SF = silk fibroin; SS = silk spidroin.

SEM was also used to observe the morphology of hWJ-MSC grown on the scaffolds for 48 h. The image (Fig. 6) showed that cells grown on 100% SF scaffold were round in shape compared to cells grown on scaffolds with spidroin silk that appeared more elongated and had spread evenly all over scaffold surface. Cells grown on silk fibroin scaffold had more rounded morphology since fibroin contains no specific amino acid sequence for cell attachment, such as RGD sequence^18,35^. Therefore, scaffold with silk spidroin was able to support better cell attachment for hWJ-MSCs. Based on SEM result, scaffold with the compositions SF 100%, SF 90% + SS 10%, and SF 85% + SS 15% would be further tested on cell viability assay.

### Scaffold composition optimization for cell proliferation

**Figure 7:**
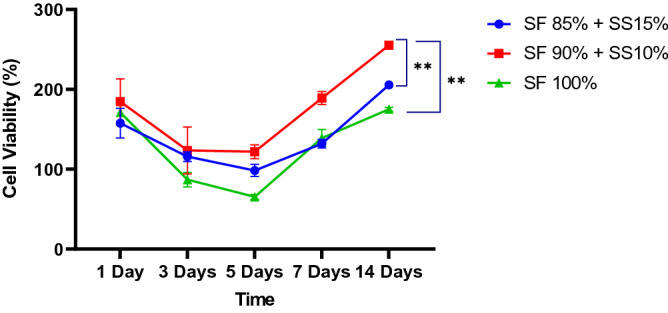
Growth curve of hWJ-MSCs grown on silk scaffold with the following composition: SF 85% + SS 15%, SF 90% + SS 10%, and SF 100% for 1, 3, 5, 7, and 14 days. Between day 5 and day 14, scaffold with the addition of SS 10% was the most optimal to support cell proliferation. ** denotes a significant difference in cell viability (*p* < 0.005). SF = silk fibroin, SS = silk spidroin.

The effect of silk scaffold composition on cell proliferation was observed through MTT assay (Fig. 7). Cells were grown for 1, 3, 5, 7, and 14 days on standard growth medium. Overall, cell viability was slightly decreased from day 1 to day 5 and then increased steadily from day 7 until day 14. Initial decrease of cell viability of cells grown on silk scaffold was also observed on *Antheraea mylitta* silk fibroin scaffold^14^ and *B.mori* silk fibroin scaffold^16,36^. This result was likely caused by cell adaptation and migration on the porous structure of the scaffold^37^. Scaffolds with the addition of spidroin silk showed higher cell viability until day 14, indicating that scaffold with silk spidroin was able to increase cell viability on the scaffold better than silk fibroin scaffold (SF 100%). Cells grown on scaffold with 90% SF + 10% SS had significantly higher growth than scaffold with 15% SS (*p* < 0.005). Based on this result, scaffold with 10% SS was the most optimal composition to support cell proliferation and should be used for chondrogenic differentiation, in comparison to SF 100% scaffold. A previous study showed that film with a blend of recombinant spidroin and silk fibroin at 10:90 had similar capacity to support cell attachment to film with higher spidroin composition^38^.

### Effect of LAA or PRP supplementation on hWJ-MSC proliferation

**Figure 8:**
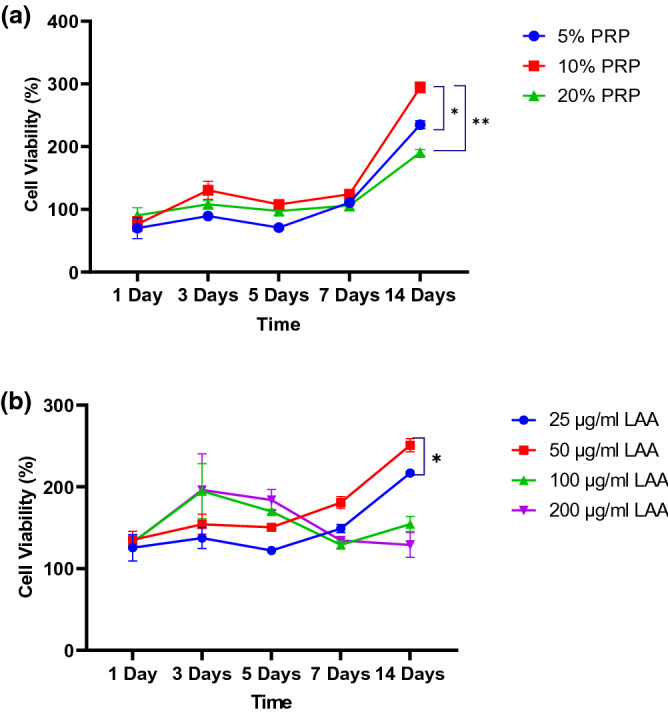
Cell viability diagram of hWJ-MSCs grown on various concentration of (**a**) PRP; PRP 5%, PRP 10%, PRP 20% and (**b**) LAA; 25 μg/ml, 50 μg/ml, 100 μg/ml, 200 μg. * denotes significant difference in cell viability (*p* < 0.05). ** denotes significant difference in cell viability (*p* < 0.01).

The optimal LAA and PRP concentrations used for hWJ-MSC chondrogenic differentiation were determined through cell viability using MTT assay. hWJ-MSC grown on medium with PRP at various concentrations (Fig. 8a) showed that cell viability was increasing until day 14. Supplementation of 10% PRP significantly had the highest cell viability for 14 days in comparison to 5% PRP and 20% PRP. Several studies showed that the addition of 10% PRP was optimal for proliferation of MSCs^7,39^ after 7 days of culture^40^. Study by Cho et al., 2011 observed an increase in DNA content after 12 days culture of MSC^41^. High concentration of PRP at 30% was shown to have no significant effect on cell proliferation in comparison to lower PRP concentration, however the mechanism is currently unclear^41^. The PRP in this study was first activated before used in cell culture. Once activated, the released growth factor contained such as PDGF, TGF-β, and FGF-2 that were able to support cell proliferation^42,43^.

LAA supplementation (Fig. 8b) showed that cell viability increased until day 14. Up to day 3, a higher dose of LAA supplementation (100 μg/ml and 200 μg/ml) was able to increase cell viability compared to the remaining treatment. However, from day 5 to day 14, the viability started to decrease. In contrast, 25 μg/ml and 50 μg/ml supplementations were able to steadily maintain cell viability from day 1 to day 14. The addition of 50 μg/ml LAA had the highest cell viability compared to the remaining concentration (*p* < 0.05). Addition of LAA at 250 mM (64,1 μg/ml) has been reported to increase proliferation of umbilical cord blood mesenchymal stem cells (UCB-MSC), while high concentration of LAA decreases cell proliferation^43^. Previous studies have also reported that supplementation of 10% PRP^16,44,45^ or 50 μg/ml LAA^11,34^ in the medium were the optimal concentration to support proliferation of MSC. Cells with higher proliferation rate before directed differentiation is known to have correlation with superior chondrogenic differentiation capacity^46^. Therefore, supplementation with 10% PRP and 50 μg/ml LAA was the most optimal concentration to support the differentiation of hWJ-MSC grown on scaffold.

### Cell attachment on silk scaffold based on RGD and integrin β1 *immunocytochemistry*

**Figure 9:**
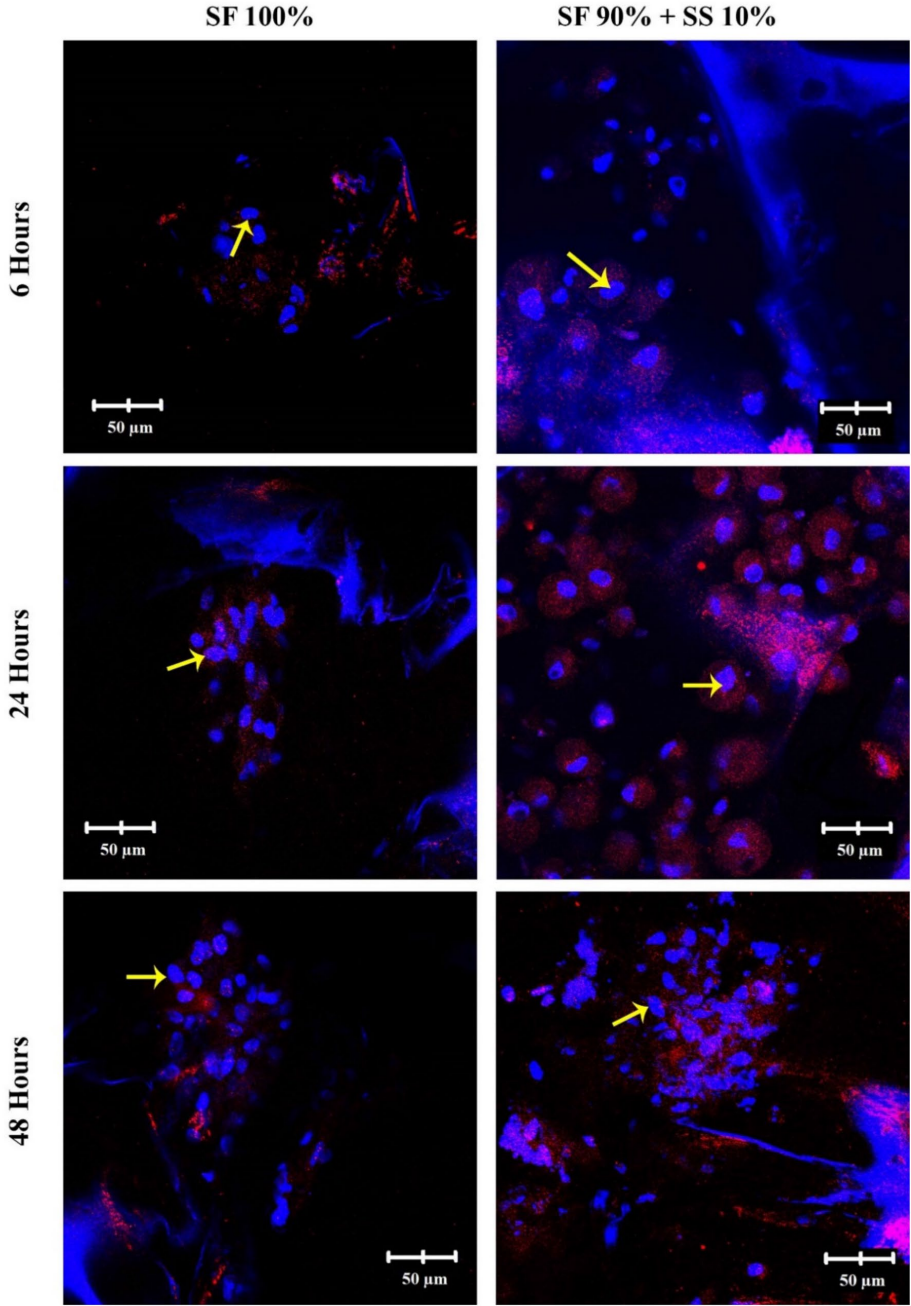
*Immunocytochemistry* (ICC) of Integrin β1 from hWJ-MSCs grown on silk fibroin-spidroin mix scaffold (SF 90% + SS 10%) and silk fibroin scaffold (SF 100%) observed under confocal microscope 6, 24, and 48 h post seeding. Integrin β1 appeared red on the images. Yellow arrows indicate the cell nuclei. SF = silk fibroin; SS = silk spidroin.

Attachment of cells on a biomaterial surface is an important step to further sustain proliferation and differentiation of cells. Integrin β1 is important in mediating early interaction between cells and matrix or biomaterial. On images of integrin β1 (Fig. 9) from hWJ-MSCs grown on scaffold without spidroin silk, SF 100% appeared to have significantly less integrin intensity (shown in red) in comparison to cells grown on SF 90% + SS 10% scaffold. The cells on scaffold with spidroin appeared to be spread all over the scaffold surface. This result was supported by SEM analysis, in which after 48 h the cells on SF 100% scaffold were forming aggregate with rounded morphology, while cells grown on SF 90% + SS 10% scaffold already appeared more elongated, with cell protrusion covering the scaffold surface. Based on the confocal images, the scaffold with silk spidroin was hypothesized to contain the specific amino acid sequence that could possibly affect the attachment of hWJ-MSC on the scaffold surface via integrin β1.

**Figure 10:**
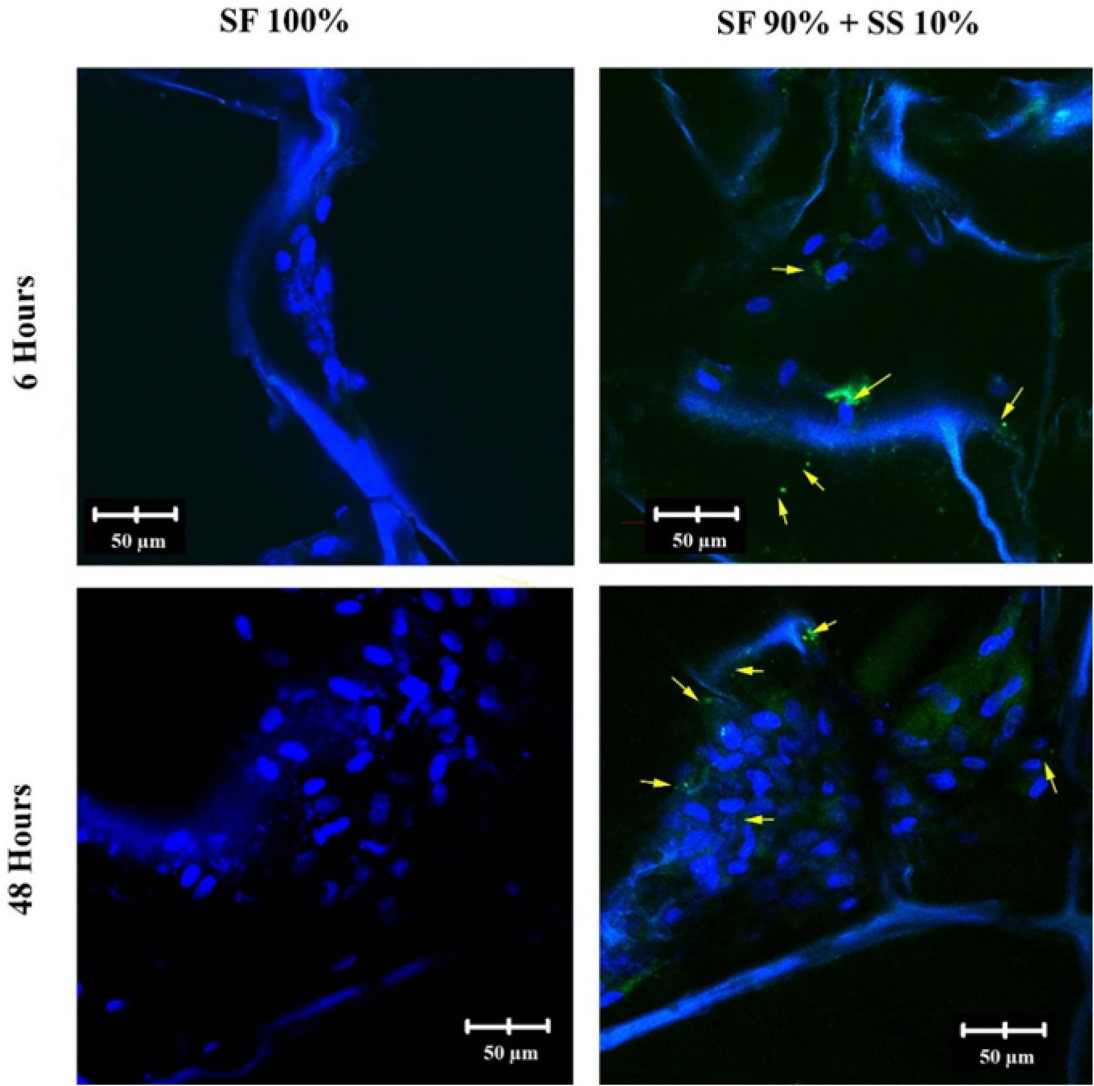
Confocal images of RGD sequence present in the scaffold with the addition of 10% silk spidroin, which appeared as small green dots pointed by the yellow arrows. SF = silk fibroin; SS = silk spidroin.

Silk produced by non-mulberry silkworm *Antheraea mylitta* is known to contain RGD sequence^47^. Scaffold produced from the silk of *A.mylitta,* was able to support attachment of rat neonatal cardiomyocyte better than silk fibroin scaffold^48^. In this study, the presence of RGD sequence on spidroin silk produced by spider *Argiope appensa* is still being investigated. Based on confocal images of RGD immunocytochemistry (Fig. 10), scaffold with spidroin silk had little green dots of RGD sequence scattered over the surface which could indicate the presence of RGD sequence in the *A.appensa* spidroin. Integrin β1 could recognize RGD sequence in the silk scaffold, thus provide more efficient cell attachment within the structure^49^. The sequence is commonly found in fibronectin, known to further activated integrin which then will interact with talin, vinculin, and actin filaments forming focal adhesion complex^50^. The complex is essential for cell survival, as it would had effect on cell polarity and migration^51,52^. Previous study observed vinculin expression on hWJ-MSCs grown on scaffold with silk spidroin, and the result showed that vinculin expression increased on scaffold with spidroin in comparison to silk fibroin scaffold.

### hWJ-MSC differentiation on silk scaffold with PRP or LAA supplementation based on GAG staining and collagen type II Immunochemistry

**Figure 11:**
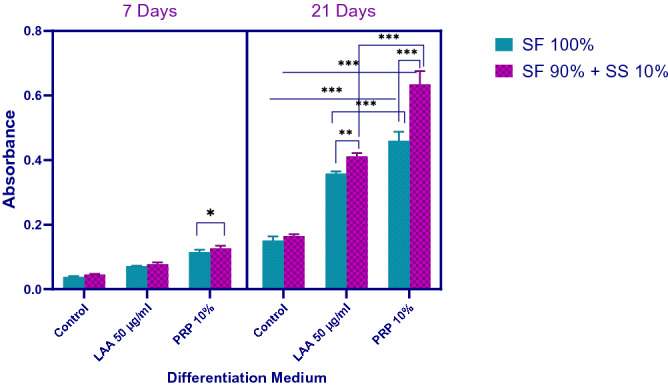
GAG accumulation of hWJ-MSC grown on silk scaffold with LAA or PRP containing differentiation medium after 7 days and 21 days (SF = silk fibroin, SS = silk spidroin, LAA = L-ascorbic acid, PRP = platelet rich plasma). * denotes significant difference (*p* < 0.05). ** denotes significant difference in cell viability (*p* < 0.01). *** denotes significant difference in cell viability (*p* < 0.001).

hWJ-MSCs were grown on scaffolds with PRP or LAA induction medium for 21 days to analyze GAG accumulation through Alcian Blue staining. The absorbance of GAG stained with Alcian Blue was measured at 650 nm (Fig. 11). Overall, cells grown on 10% (v/v) PRP medium had higher GAG accumulation in comparison to 25 μg/ml LAA medium and control. Cells grown on scaffold 90% SF + 10% SS had higher GAG accumulation compared to cells grown on 100% SF scaffold with PRP or LAA supplementation. Based on these results, 10% (v/v) PRP supplementation was the most optimal treatment to support chondrogenic differentiation, and incorporation of 10% silk spidroin into the scaffold also influenced the amount of GAG accumulation.

**Figure 12:**
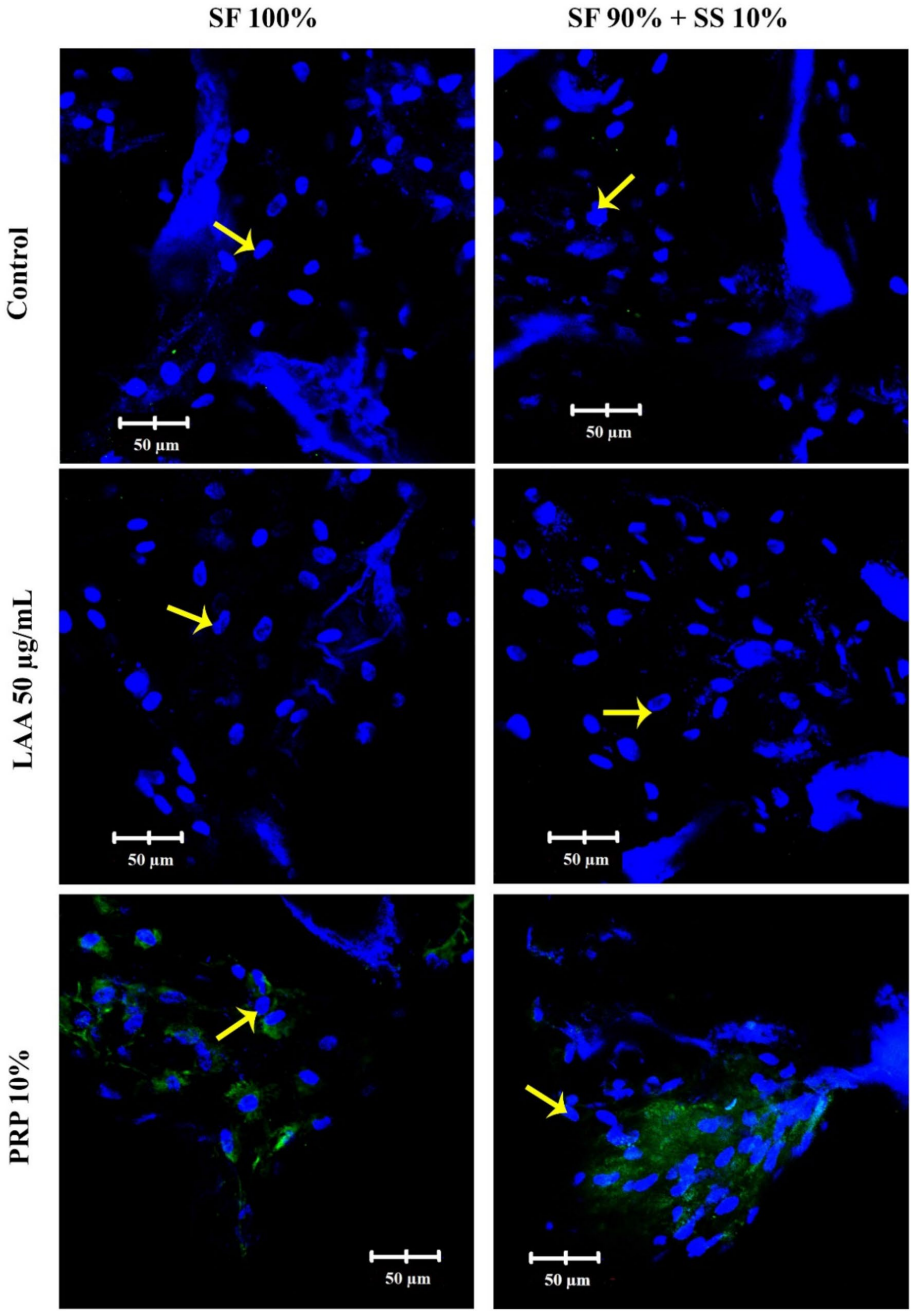
*Immunocytochemistry* (ICC) of collagen type II in hWJ-MSCs grown on silk fibroin-spidroin mix scaffold (SF 90% + SS 10%) and silk fibroin scaffold (SF 100%), cultured on differentiation medium with FBS 10% (control), LAA 50 µg/mL, and PRP 10%. The images were observed under confocal microscope after 7 days of culture. Collagen type II appeared green on the images. Yellow arrows indicate the cell nuclei. SF = silk fibroin; SS = silk spidroin.

**Figure 13:**
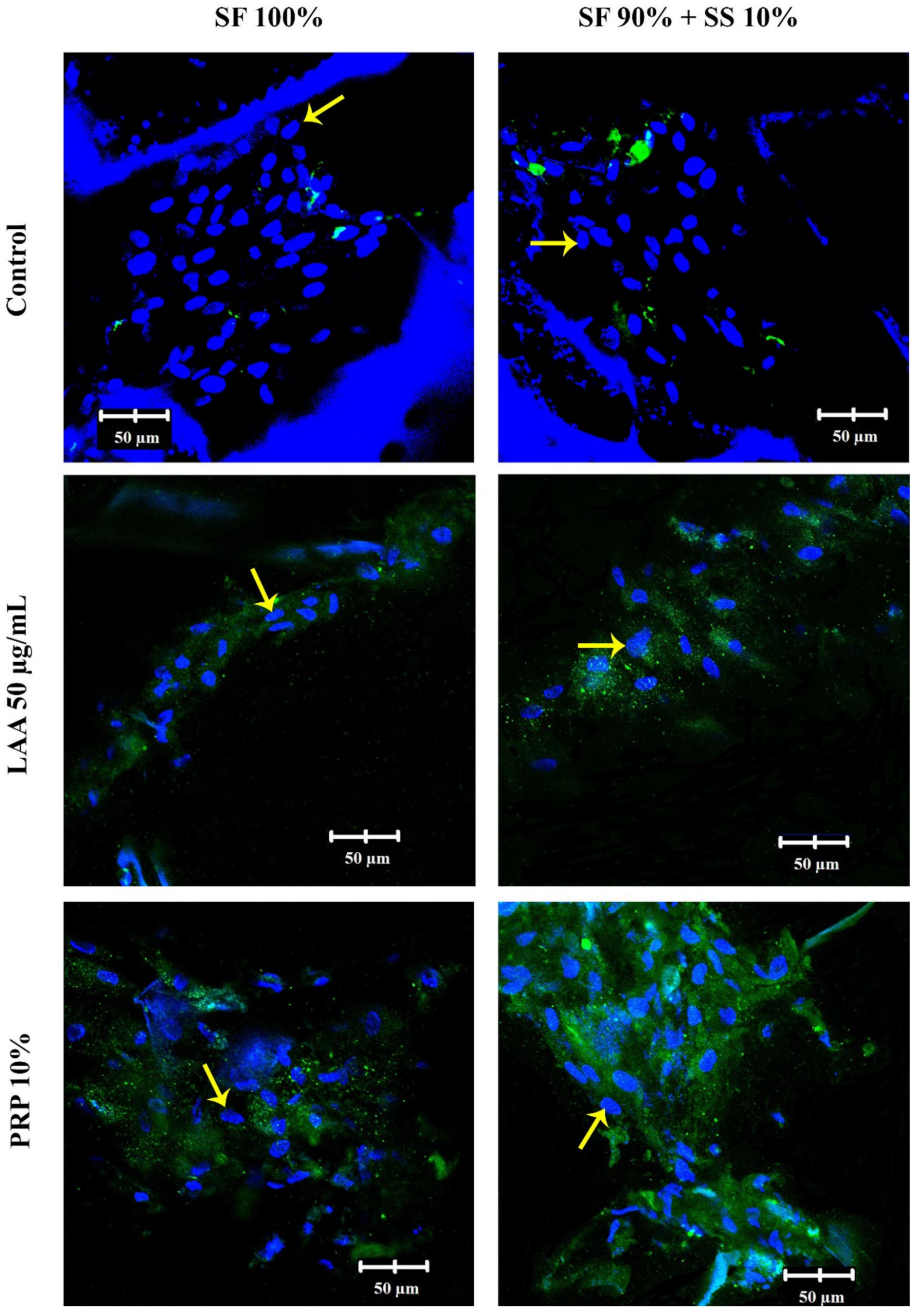
*Immunocytochemistry* (ICC) of collagen type II in hWJ-MSCs grown on silk fibroin-spidroin mix scaffold (SF 90% + SS 10%) and silk fibroin scaffold (SF 100%), cultured on differentiation medium with FBS 10% (control), LAA 50 µg/mL, and PRP 10%. The images were observed under confocal microscope after 21 days of culture. Collagen type II appeared green on the images. Yellow arrows indicate the cell nuclei. SF = silk fibroin; SS = silk spidroin.

Collagen type II is the specific component produced by cells during chondrogenic differentiation, and in this research was observed through a confocal microscope after differentiation for 7 and 21 days. On day 7 (Fig. 12), differentiation of hWJ-MSCs with PRP medium had already started to produce collagen type II, while those with LAA medium had not. Cells grown on scaffold SF 90% + SS 10% with PRP 10% medium appeared to produce more of collagen type II in comparison to cells grown on fibroin scaffold (SF 100%). On day 21 (Fig. 13), collagen II produced from hWJ-MSCs grown on SF 90% + SS 10% with PRP 10% medium were the highest.

The effect of PRP on MSC chondrogenesis has been reported in several studies. Addition of PRP 10% into differentiation medium was reported to be the most optimal concentration to induce chondrogenesis in hADSC (human adipose-derived stem cells)^16,53,54^ by increasing the synthesis of GAG and collagen type II into the surrounding ECM after 21 days. Activated PRP releases several growth factors, with TGF-β1 (transforming growth factor beta) being one of the highest in concentration^55,56^. TGF-β1 binds to TGF receptor, causing signaling cascade involving Smad 2/3 and 4 protein which then translocate into the nucleus and interacting with transcription factor Sox 9. Sox 9 is important in regulating expression of collagen type II and GAG^54,57^.

Overall, in this research scaffold with silk spidroin (SF 90% + SS 10%) was able to support chondrogenic differentiation of hWJ-MSCs significantly better in comparison to silk fibroin scaffold (SF 100%) as shown in collagen type II ICC and GAG accumulation. Based on SEM analysis and Integrin β1 ICC, it was speculated that the spidroin silk collected from *Argiope appensa* contained a specific amino acid sequence, such as RGD, which was recognized by cells to attach on a biomaterial surface or ECM. Previous research on silk produced from *A.mylitta*, which is known to contain RGD sequence, showed that it was able to support chondrogenic and osteogenic differentiation better than silk fibroin scaffold^14,58^. In this research the presence of RGD sequence in the *A.appensa* silk were observed through confocal images, which was incorporated into the silk fibroin-spidroin mix scaffold. The addition of silk spidroin has improved the mechanical properties of the porous scaffold, which is an important factor in cartilage tissue engineering. Scaffold with 10% SS also provided better surface for cell attachment, an important early step for cell survival, proliferation, and differentiation with the addition of PRP.

## Materials and Methods

### Isolation and primary culture of human Wharton’s Jelly Mesenchymal stem cells (hWJ-MSC)

Human umbilical cord samples were obtained from caesarean section deliveries at Rumah Sakit Khusus Ibu dan Anak (RSKIA), Bandung with approval from the Health Research Ethics Committee, Faculty of Medicine, Padjajaran University (Number: 09/UN6.KEP/EC/2020). The participants were informed and agreed on the sampling of the umbilical cord. All methods in this research were in accordance with relevant guidelines and regulations. Explant method was used to isolate primary hWJ-MSC from the umbilical cord sample^32^. 10–15 cm of umbilical cord was washed with sterile PBS, and then the arteries were removed from the Wharton’s Jelly part. The tissue was cut into about 0.5–1 cm pieces, then attached to 100 mm tissue culture plate. Once attached, 5 mL of growth medium were carefully added around the tissues. Growth medium was Dulbecco’s Modified Eagle’s Medium (DMEM) supplemented with 10% fetal bovine serum and 1% antibiotic–antimycotic. Culture cells were incubated at 37 °C with 5% CO_2_, and the medium was replaced every 2 days. After about 10–12 days, when cells with fibroblast-like morphology migrated from the explant, the tissues were removed. Once the primary culture (P0) reached 80% confluency, the cells were expanded until passage 5 (P5).

### Characterization of hWJ-MSC

#### Multipotency assay

hWJ-MSCs P5 were grown on 24 well plate (2 × 10^4^ cells/well) for multipotency assay. Once the confluency reached about 80% the growth medium was replaced with adipogenic medium (StemPro Adipogenesis Differentiation Kit), chondrogenic medium (StemPro Chondrogenesis Differentiation Kit), and osteogenic medium (StemPro Osteogenesis Differentiation Kit). The culture was incubated for 21 days at 37 °C and 5% CO_2_. After 21 days, the remaining medium was washed with PBS 3 times and the cells were fixed with 4% formaldehyde. Cells were stained with Oil Red O to stain lipid droplet formation, Alcian Blue to stain glycosaminoglycan (GAG) accumulation, or Alizarin Red to stain calcium deposits. Staining results were observed under an inverted microscope.

#### MSC specific surface marker

Flow cytometry analysis of a specific mesenchymal surface marker was performed using Human MSC Analysis Kit (BD Bioscience). The kit contains hMSC positive cocktail (CD90 FITC, CD105PerCP, and CD73APC) and MSC negative cocktail (CD34 PE, CD11 PE, CD19, CD45, and HLA-DR). hWJ-MSCs passage 5 was tripsinized and resuspended in 1 ml buffer, then each 100 ul of the suspension was stained with 5 ul of the positive cocktail, negative cocktail, or isotype. Samples were washed with staining buffer and resuspended with 500 ul staining buffer, and then the flow cytometry was performed with BD FACSAria II and analyzed with software BD FACSDiva 8.0.2.

### Preparation of silk fibroin-spidroin scaffold

Two types of scaffold were used: silk fibroin scaffold (SF) and silk fibroin mixed with silk spidroin scaffold (SF + SS) with various compositions (Table 2). Scaffolds were fabricated using a modified salt leaching method^24^. Silk fibroin (SF) fibers collected from a *Bombyx mori* (CV. Wisata Imu Sutra, Bandung, West Java, Indonesia) cocoon were degummed to remove the sericin protein by heating the silkworm cocoon in a solution of 0.05 (wt/v%) NaHCO_3_ for 1 hour^59^. Silk spidroin (SS) were collected from spider *Argiope appensa*. *A. appensa* were caught around Lembang, West Java. The spiders were placed and fed regularly in a wooden box with lid covered with thin meshed fabrics. Dragline silk produced on the box were collected/rolled manually using clean tools without harming the spiders. The degumming process was not carried out on silk spidroin (SS) fibers*.* Scaffolds were prepared with the following composition: SF 100% (w/w), SS 15% (w/w) + SF 85% (w/w), SS 10% (w/w) + SF 90% (w/w).

**Table 2:** Weight of silk fibroin and silk spidroin to produce porous silk fibroin-spidroin mix scaffold.

Scaffold composition	Silk fibroin (gram)	Silk spidroin (gram)
SF 100%	0.06	–
SF 95% + SS 5%	0.057	0.003
SF 90% + SS 10%	0.054	0.006
SF 85% + SS 15%	0.051	0.009
SF 80% + SS 20%	0.048	0.012

A total of 0.06 g of silk was dissolved in 500 µL of 8 wt% CaCl_2_-formic acid solution (PT. Bratachem, Bandung) at room temperature. The solution was then poured into a polystyrene mold and mixed with 2.5 g of 500 µm NaCl particles. NaCl particles create the pores in scaffolds. The NaCl-silk mixture was dried on a fume hood overnight. Once the scaffold was formed, it then was soaked in 70% ethanol for 30 min. The scaffold was then immersed in distilled water for 3 days to remove the NaCl. The scaffold was released from the base of the mold and cut into 5 × 5 mm size.

### Scaffold scanning electron microscope (SEM) analysis

SEM was utilized to observe the formation of interconnected pore, pore size, and distribution of hWJ-MSCs inside the scaffold. SEM analysis was also used, to observe the morphology of hWJ-MSC on the scaffold surface. hWJ-MSCs were grown on the silk scaffolds for 48 h at 37 °C; 5% CO_2._ The samples were fixed with 100 µl of 2.5 glutaraldehyde in 0.1 M cacodylate buffer, incubated for 24 h at 4 °C, dehydrated with serial alcohol, and freeze dried for 3 h. Samples were coated with gold and observed under SEM (SU 3500; Hitachi, Krefeld, Germany; Center of Advanced Science ITB).

### Fourier transform infared (FTIR) analysis

FTIR spectroscopy was necessary in analyzing structure of the scaffolds, in this current study FTIR was performed on wet and dry unprocessed silk fibroin and spidroin fiber as comparison to scaffold with fibroin and spidroin mixed in. FTIR spectroscopy was recorded between 1000 cm^−1^ and 4000 cm^−1^ (Shimadzu IR Prestige-21, Japan).

### Contact angle and water uptake measurement

Hydrophilicity of the silk fibroin and silk spidroin mix scaffold was observed by measuring contact angle and water uptake capacity of the scaffolds. The contact angle was measured by dropping 10 µl of distilled water onto the surface. The angle formed was captured after 10 s using portable microscope and measured using application (Dino-Lite, Taiwan). Scaffold capacity to absorb water/ water uptake was measured by submerging previously weighed dry scaffold (W_0_) into distilled water for 24 h at room temperature. Any excess water was removed using paper towel, and the wet scaffold was weighed again (W_1_) to obtain scaffold water uptake capacity.

$${\text{Water}}\;{\text{uptake}} = \left( {{\text{W}}_{1} {-}{\text{W}}_{0} } \right)/{\text{W}}_{0} \times 100\%$$

### Mechanical testing (compressive)

The cylindrical scaffold of fibroin and spidroin silk with 13 mm in diameter and 6 mm in thickness was tested with compressive machine (TENSILON Universal testing machine). The scaffold was in dry condition with drying in ambient temperature for 72 h. The machine pressed the scaffold from 6 to 2 mm (about 60% strain) with compressive speed at 0.1 mm/min. The result was then plotted to obtain the compressive strength and modulus of elasticity of the scaffold.

### Growth of hWJ-MSCs on silk scaffold

The growth of hWJ-MSCs grown on silk scaffold was analysed with ([3-(4,5-dimethylthiazol-2-yl)-2,5-diphenyltetrazolium bromide]) MTT cytotoxicity assay. 10^5^ hWJ-MSCs were grown with standard growth medium on the scaffolds, with the following compositions: 100% SF, 90% SF + 10% SS, and 85% SF + 15% SS for 1, 3, 5, 7, and 14 days. As a control, 10^5^ hWJ-MSCs were grown on 96 well plate without scaffold. After 1, 3, 5, 7, and 14 days the remaining medium on each well was replaced with 10 µl MTT reagent in 100 µl growth medium and incubated in the dark for 4 h at 37 °C incubator with 5% CO_2_. The MTT reagent was discarded, and 100 µl of DMSO were added on each well and incubated at room temperature for 5 min to dissolve the formazan crystal. Absorbance of the solution was read using a microplate reader at 570 nm (n = 3).

### Optimization of PRP and LAA concentration as chondrogenesis inducer

The effect of PRP and LAA on hWJ-MSC proliferation was observed through MTT assay. PRP concentration in the induction medium was 5% (v/v), 10% (v/v), and 20% (v/v). LAA concentration in the medium was 25 µg/ml. 50 µg/ml, 100 µg/ml, and 200 µg/ml. 2 × 104 cells were grown on 96 well plates for 1, 3, 5, 7, and 14 days. The remaining medium on each well was replaced with 10 µl MTT reagent in 100 µl growth medium and incubated in the dark for 4 h at 37 °C incubator with 5% CO_2_. The MTT reagent was discarded, and 100 µl of DMSO were added on each well and incubated at room temperature for 5 min to dissolve the formazan crystal. Absorbance of the solution was read using a microplate reader at 570 nm (n = 3).

### Integrin β1 immunocytochemistry

5 × 10^5^ of hWJ-MSCs were grown on scaffold SF 100% and SF 90% + 10% for 6, 12, and 48 h. The cells were fixated using serial methanol-DMEM (50%, 70%, and 80%) for 5 min at − 20 °C, and further fixated with 100% methanol for 20 min at − 20 °C. The fixated cells were permeabilized using 0.05% Tween-20 in PBS for 20 min and blocked with 3% BSA (*bovine serum albumin*) in PBS for 60 min. A primary antibody against integrin β1 (PA5-29606, ThermoFisher Scientific) was added and followed by overnight incubation at 4 °C. Any remaining primary antibodies were washed with PBS three times. The cells were incubated with secondary antibody (Alexa Fluor 647, Abcam) for 2 h. 2,5 µg/ml DAPI (4′,6-diamidino-2-phenylindole) was added to stain cell nucleus. The images were observed using a confocal microscope (Olympus Fv1200) at three different points^54^.

### RGD sequence immunocytochemistry

5 × 10^5^ of hWJ-MSCs were grown on scaffold SF 100% and SF 90% + 10% for 6 and 48 h. The cells were fixated using serial methanol-DMEM (50%, 70%, and 80%) for 5 min at − 20 °C, and further fixated with 100% methanol for 20 min at − 20 °C. The fixated cells were permeabilized using 0.05% Tween-20 in PBS for 20 min and blocked with 3% BSA (*bovine serum albumin*) in PBS for 60 min. A primary antibody against integrin RGD (ab224465, Abcam) was added and followed by overnight incubation at 4 °C. Any remaining primary antibodies were washed with PBS three times. The cells were incubated with secondary antibody (Alexa Fluor 488, Abcam) for 2 h. 2,5 µg/ml DAPI (4′,6-diamidino-2-phenylindole) was added to stain cell nucleus. The images were observed using a confocal microscope (Olympus Fv1200) at three different points^54^.

### Alcian Blue staining to quantify GAG content

Alcian Blue staining was used to quantify GAG accumulation of the cells grown on the silk scaffolds. hWJ-MSCs were grown until the end of passage 4. Once the confluency reached 80%, the cells were trypsinized. 5 × 10^5^ cells were resuspended in 20 µl medium and seeded onto the scaffolds and then incubated at 37 °C 5% CO_2_ for 3 h to allow attachment of cells onto the scaffold surface. After 3 h, the constructs were moved into non-treated 24 well plate, and 500 ul induction medium were added. The culture was maintained for 21 days, and the medium was replaced every 2 or 3 days. After 21 days, the remaining medium was discarded and the culture was washed three times with PBS. The culture was fixed with acetone-methanol (50%: 50%, v/v) for 3 min at 4 °C. The fixative solution was removed and 1% (v/v) Alcian Blue in 3% (v/v) acetic acid was added into the construct and incubated for 30 min at room temperature. The remaining stain was washed with 3% acetic acid and deionized water, then 1% (w/v) of SDS was added to the well and incubated on a shaker for 30 min. Absorbance reading of dissolved GAG was done with a microplate reader at 605 nm.

### Collagen type II ICC

5 × 10^5^ of hWJ-MSCs were grown on scaffold SF 100% and SF 90% + 10%, with the addition of LAA or PRP differentiation medium for 7 and 21 days. The cells were fixated using serial methanol-DMEM (50%, 70%, and 80%) for 5 min at − 20 °C, and further fixated with 100% methanol for 20 min at − 20 °C. The fixated cells were permeabilized using 0.05% Tween-20 in PBS for 20 min and blocked with 3% BSA (*bovine serum albumin*) in PBS for 60 min. A primary antibody against collagen type II (ab34712, Abcam) was added and followed by overnight incubation at 4 °C. Any remaining primary antibodies were washed with PBS three times. The cells were incubated with a secondary antibody (Alexa Fluor 488, Abcam) for 2 h. 2.5 µg/ml DAPI (4′,6-diamidino-2-phenylindole) was added to stain cell nucleus. The images were acquired and observed at three random different points using confocal microscope (Olympus Fv1200) and Fluoview software.

### Statistical analysis

Evaluation of hWJ-MSC viability and GAG were performed using two-way analysis of variance (ANOVA). Post-hoc analysis was performed via Tukey’s multiple comparison test. All values are expressed as mean and represent three independent experiments. All analysis was carried out using software GraphPad Prism version 8.4.1 for Windows (GraphPad Software, San Diego CA).

## Supplementary information

Supplementary information.

Supplementary information.
